# Hitchhiking with forests: population genetics of the epiphytic lichen *Lobaria pulmonaria* in primeval and managed forests in southeastern Europe

**DOI:** 10.1002/ece3.341

**Published:** 2012-08-01

**Authors:** Christoph Scheidegger, Peter O Bilovitz, Silke Werth, Ivo Widmer, Helmut Mayrhofer

**Affiliations:** 1 Swiss Federal Research Institute WSLZürcherstrasse 111, 8903, Birmensdorf, Switzerland; 2 Institute of Plant Sciences, Department of Systematic Botany and Geobotany, Karl-Franzens-University GrazHolteigasse 6, 8010, Graz, Austria

**Keywords:** Conservation biology, lichen ecology, phylogeography, population genetics, primeval forests

## Abstract

Availability of suitable trees is a primary determinant of range contractions and expansions of epiphytic species. However, switches between carrier tree species may blur co-phylogeographic patterns. We identified glacial refugia in southeastern Europe for the tree-colonizing lichen *Lobaria pulmonaria*, studied the importance of primeval forest reserves for the conservation of genetically diverse populations and analyzed differences in spatial genetic structure between primeval and managed forests with fungus-specific microsatellite markers. Populations belonged to either of two genepools or were admixed. Gene diversity was higher in primeval than in managed forests. At small distances up to 170 m, genotype diversity was lower in managed compared with primeval forests. We found significant associations between groups of tree species and two *L. pulmonaria* genepools, which may indicate “hitchhiking” of *L. pulmonaria* on forest communities during postglacial migration. Genepool B of *L. pulmonaria* was associated with European Beech (*Fagus sylvatica)* and we can hypothesize that genepool B survived the last glaciation associated within the refuge of European Beech on the Coastal and Central Dinarides. The allelic richness of genepool A was highest in the Alps, which is the evidence for a northern refuge of *L. pulmonaria*. Vicariant altitudinal distributions of the two genepools suggest intraspecific ecological differentiation.

## Introduction

Ecological processes at various spatial and temporal scales leave their genetic footprints in populations. Congruent genetic pattern between coexisting or interacting species has been attributed to either, or a combination of three processes, that is, (1) congruent response to shared environmental change, (2) joint dispersal, or (3) nested habitat requirement such as the presence of a carrier tree for an obligate epiphytic species. First, congruent response to environmental change has been studied by a phylogeographic approach for a broad diversity of organisms (Bermingham and Moritz 1998; Taberlet et al. 1998; Avise 2000; Arbogast and Kenagy 2001; Knowles and Maddison 2002; Soltis et al. 2006; Carstens and Richards 2007; Printzen 2008; Hewitt 2011) and guilds such as mesic forest organisms were reported to have responded in a concerted manner to environmental change, often from incongruent ancestral distributions (Carstens and Richards 2007).

Second, congruence in genetic patterns may be the result of joint dispersal and joint range expansion of species with strong biological interactions, such as epiphytes and the tree populations they depend on. High levels of congruence can be expected from host and parasite phylogeography (Beibl et al. 2007; Nieberding et al. 2008; Miles et al. 2009; Stone et al. 2009; Stefka et al. 2011), although it is not always perfect (Criscione et al. 2005). Thus, the spatial genetic structure of interacting taxa is influenced by ecological as well as evolutionary factors: for example, geographic distance between populations, adaptation to local environmental conditions, and reciprocal specificity of the interaction. In lichens, congruence in genetic patterns relates to the fungal reproductive mode. Species reproducing sexually via small ascospores are generally thought to be very efficient dispersers, while clonal dispersal can lead to substantial genetic structure (Wagner et al. 2005, 2006; Werth et al. 2006; Dal Grande et al. 2012). In the widespread, although regionally rare and threatened tree-colonizing lichen species *Lobaria pulmonaria* (L.) Hoffm. via clonal, symbiotic propagules accounts for more than 70% of reproduction within populations (Dal Grande et al. 2012). Consequently, a highly congruent genetic population structure of the fungal and algal symbionts was found in this species both at the landscape and regional scales (Widmer 2009; Werth and Scheidegger 2012). Moreover, we would expect this species to show some congruence with the populations of the tree species it has been associated with for an extended time period. However, in the arctic alpine *Cetraria aculeata* which forms an association with a *Trebouxia* photobiont, the photobiont shows a climatic distribution and the mycobiont shows strong geographic structure (Fernandez-Mendoza et al. 2011).

Third, phylogeographic congruence may be the result of an epiphyte's dependence on specific carrier trees as habitat. Narrow carrier tree spectra are known for a variety of lichen species, especially in tree species with extreme pH or microhabitat characteristics such as deep cortical crevices (Stofer et al. 2008). If the association with a particular tree species is stable over several lichen generations, the associated lichen species (which notably includes the fungal and the algal symbionts) may show phylogeographic congruence.

**Figure 1:**
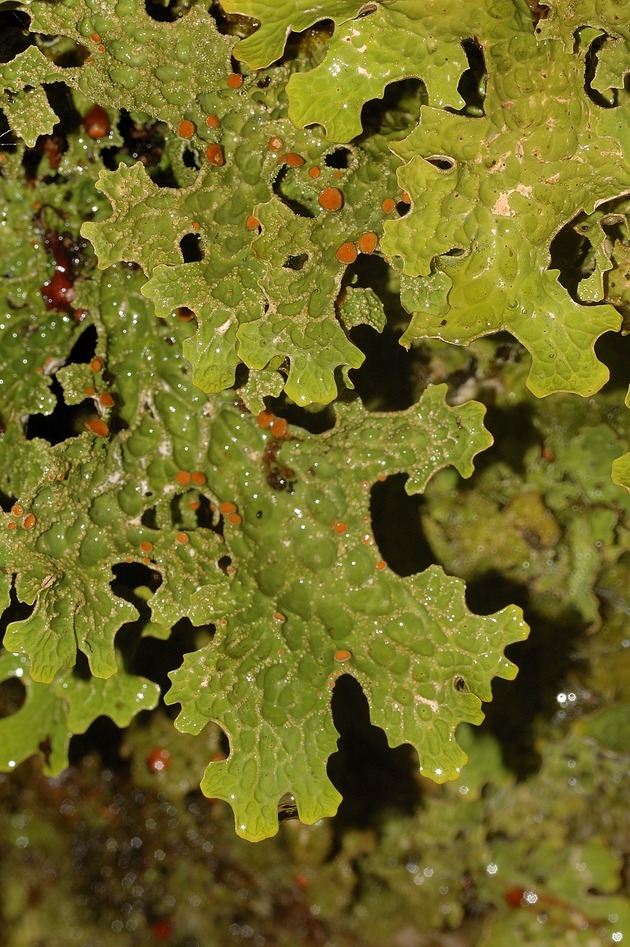
*Lobaria pulmonaria* is a rare and threatened tree-colonizing lichen species. The species is heterothallic and has a complex reproductive strategy that includes sexual reproduction of the fungal partner with ascospores which are formed in brownish-red apothecia, and vegetative propagules where the fungal and algal partner form symbiotic propagules in small cracks (soralia) scattered on the thallus.

*Lobaria pulmonaria* is a tripartite, nitrogen fixing foliose lichen (Fig. 1) which has a narrow, subacidophytic pH range (4.1–5.6) (Barkman 1958; Wirth 1995) and the lichen's carrier tree range is thus restricted to a subset of the European forest-tree species including *Abies alba* and *A. cephalonica*, *Acer pseudoplatanus, Castanea sativa, Fagus sylvatica*, and *F. orientalis, Fraxinus excelsior* and *F. ornus, Quercus* sp. div, but excluding *Picea, Pinus*, and *Tilia*. *Lobaria pulmonaria* is a widespread, although regionally threatened epiphytic lichen with documented old-growth forest requirements (Rose 1976, 1992; Scheidegger and Werth 2009). In all climatic regions intensive forest management leads to regional extinctions of this species and only a substantial reduction of tree harvest can possibly maintain viable populations of *L. pulmonaria* in managed forests. Changing tree species composition, light availability at the lichen's habitat (Pannewitz et al. 2003), and altered spatio-temporal patterns of the carrier trees (Wagner et al. 2006; Scheidegger and Werth 2009; Jüriado et al. 2011) are known effects of forest management that can lead to the decline of *L. pulmonaria*. Continuing multifunctional forest management in wooded pastures maintained a *L. pulmonaria* population with a relatively high abundance, although with a reduced genetic diversity compared with old-growth forests (Jüriado et al. 2011). These authors suggested that the altered spatial pattern of the carrier trees has changed due to agro-forestry since medieval times, although wooded meadows with a more open forest structure due to grazing revealed better preserved *L. pulmonaria* populations than stands managed purely for timber production. However, it is still unclear, if sustainably managed forests harbor levels of genetic diversity that are comparable to primeval forests. With the current efforts to optimize forest management for biodiversity conservation, reference values of biodiversity, including genetic diversity are important to know and to set in relation with efforts made in biodiversity conservation in managed forests.

Our study aimed at elucidating (1) the phylogeography of *L. pulmonaria* in southeastern Europe, (2) the importance of primeval forest reserves for the conservation of large and genetically diverse populations compared with populations surviving in managed forests, and (3) to study differences in the spatial genetic structure between primeval and managed forests.

## Material and Methods

### Study area

We sampled 24 populations of *L. pulmonaria* from the Austrian Alps (4), Dinarides from Slovenia (2), Bosnia and Herzegovina (3) and Montenegro (9), Strandzha Mountains (3) and the Central Balkan Mountains (1) of Bulgaria and the Pindos Mountains in Greece (2) (Table 1). Wherever available we included populations from primeval forest reserves and managed forests. In the Eastern Alps the Rotwald (AU6) is the largest primeval forest reserve (Bilovitz 2007) and the locality Feistritzklamm (AU8) is the lowest known locality of *L. pulmonaria* (Bilovitz 2002). In the primeval forest reserve Rajhenavski Rog of the Slovenian Dinarides (Bilovitz et al. 2011) *L. pulmonaria* was scarce but the species was rather frequent in the vicinity of the reserve (SL8) in managed stands. Perućica in Bosnia and Herzegovina and Biogradska gora in Montenegro are among the largest and best preserved primeval forests in Europe (Parviainen 2005; Bilovitz et al. 2009; Bilovitz and Mayrhofer 2010). In Bulgaria all populations were collected in managed forests, although some were close to primeval forest stands (Raev et al. 2005; Spier et al. 2008).

**Table 1:** Localities, collectors, forest stand conservation status and main substrate of studied *Lobaria pulmonaria* populations. Collectors (Coll.): A. Atanassova (1), P. Bilovitz (2), A. Drescher (3), L. Lökös (4), H. Mayrhofer (5), V. Pertsalis (6), D. Stešević (7). Conservation status (Cons.): protected primeval forest (1), managed forest (2). Fertility (Fert.) in percent of fertile specimens of investigated samples. Substrate (Subst.): *Acer campestre* (1), *A. pseudoplatanus* (2), *Carpinus orientalis* (3), *Castanea sativa* (4), *Fagus sylvatica* (5), *Fraxinus excelsior* (6), *Quercus frainetto* (7), *Quercus* sp. (8)

Code	Country	Locality	Longitude	Latitude	Altitude (m)	Coll.	Cons.	Fert.	Subst.
AU5	Austria	Northern Alps, Türnitzer Alps, NE of Mariazell, Rechengraben	15°21′E	47°47′N	820–860	2	2	15	2
AU6	Austria	Northern Alps, Ybbstaler Alps, W of Mariazell, Rotwald, “GroßerUrwald”	15°05′E	47°46′N	960–990	2	1	13	5
AU7	Austria	Northern Alps, Ennstaler Alps, NW of Hieflau, Tamischbachgraben	14°41′E	47°38′N	690–840	2	2	38	2
AU8	Austria	Central Alps, Fischbacher Alps, NATURA 2000 area Feistritzklamm/Herberstein	15°48′E	47°13′N	370–380	2	2	13	6
SL8	Slovenia	Central Dinarides, KocevskiRog, road to the virgin forest RajhenavskiRog	15°00′E	45°41′N	690–740	2	2	25	2
SL9	Slovenia	Coastal Dinarides, NotranjskiSnežnik, LoskaDolina, near BabnoPolje	14°31′E	45°39′N	770–790	2	2	4	2
BH1	Bosnia and Herzegovina	Central Dinarides, Maglić, Sutjeska NP, virgin forest Perućica	18°41′E	43°18′N	1190–1240	2	1	20	5
BH2	Bosnia and Herzegovina	Central Dinarides, Maglić, Sutjeska NP, virgin forest Perućica	18°42′E	43°19′N	1060–1180	2	1	28	5
BH3	Bosnia and Herzegovina	Central Dinarides, Maglić, Sutjeska NP, virgin forest Perućica, near stream Perućica	18°42′E	43°18′N	1030–1070	2	1	53	2
MN1	Montenegro	Central Dinarides, Bjelasica, Biogradska gora NP, road to Dolovi above Biogradskojezero	19°37′E	42°54′N	1330–1550	2	1	3	5
MN2	Montenegro	Central Dinarides, Bjelasica, Biogradska gora NP, Biogradskojezero	19°35′E	42°54′N	1100–1130	2	1	0	5
MN3	Montenegro	Central Dinarides, Bjelasica, Biogradska gora NP, path to KatunGoles above Biogradskojezero	19°36′E	42°53′N	1220–1290	2	1	0	5
MN4	Montenegro	Central Dinarides, Bjelasica, Biogradska gora NP, delta of Biogradska rijeka	19°36′E	42°53′N	1100–1110	2	1	25	5
MN5	Montenegro	Central Dinarides, Bjelasica, road to KatunVranjak above Jezerine	19°38′E	42°50′N	1440–1610	2	2	0	5
MN6	Montenegro	Central Dinarides, Maganik, Mrtvica canyon	19°20′E	42°44′N	240–370	7	2	3	1,3
MN7	Montenegro	Central Dinarides, Ljubišnja, Tara canyon, Lever Tara	19°17′E	43°09′N	670–710	7	2	11	5
MN8	Montenegro	Coastal Dinarides, Rumija, Livari, near crossing to GornjaBriska	19°13′E	42°07′N	480	5	2	0	4
MN9	Montenegro	Coastal Dinarides, Rumija, S above Livari along the path to the peak of Rumija	19°12′E	42°06′N	980–1050	1,3	2	3	5
BG1	Bulgaria	Strandzha mountain massif, forest above Marina reka reserve, along the road between the villages Izgrev and Bulgari	27°46′E	42°07′N	240–250	4	2	13	8
BG2	Bulgaria	Strandzha mountain massif, road between Grammatikovo and malkoTarnovo, at Kachulskydol	27°37′E	42°01′N	130–140	4	2	14	8
BG3	Bulgaria	Central Balkan mountain range, reserve Boatin	24°15′E	42°49′N	920–1000	1	2	0	5
BG4	Bulgaria	Strandzha mountain massif, reserve Silkosya	27°44′E	42°05′N	270–310	1	2	0	7
GR9	Greece	Pindos mountain range, near Metsovo, village Chrysovitsa	21°04′E	39°47′N	1370–1420	6	2	0	5
GR10	Greece	Pindos mountain range, near Metsovo, road from Anilio to Haliki	21°11′E	39°43′N	1300–1570	6	2	14	5

### Sampling

Small fragments of *L. pulmonaria* were sampled from 20 trees from forest stands of approximately 3 ha. From the geographic center of a population we sampled 10 nearest neighbor trees colonized by *L. pulmonaria*. Additionally, 10 trees on a circle with radius of about 150 m from the plot center were sampled. From each tree three thallus fragments were collected from different parts of the trunk. Fragments had no necrotic parts and were without visible parasites or fruit bodies and measured up to 7 × 5 cm. Samples were air dried and kept frozen at −20°C until further treatment.

### Molecular analysis

Total DNA was isolated from cleaned and lyophilized lobe tips of *L. pulmonaria* using the DNeasy 96 plant kit (Qiagen, Hilden, Germany) according to the manufacturer's protocol. Eight unlinked, fungal microsatellite loci, LPu03, LPu09, LPu15, LPu23, LPu24, LPu25, LPu28, (Walser et al. 2003, 2004; Widmer et al. 2010) and MS4 (Dal Grande et al. 2012) were used. Fragment lengths of a first multiplex were determined on a 3130 Genetic Analyzer, while those of a second multiplex were analyzed on a 3730 DNA Analyzer (Applied Biosystems, Rotkreuz, Switzerland). Genotyping took place using GENEMAPPER 3.7 (Applied Biosystems) using ROX-500 (multiplex 1) or LIZ-500 (multiplex 2; Applied Biosystems) as internal size standards (Widmer et al. 2010).

### Statistical analysis

For the purpose of inferring the number of genepools across the study region, assigning *L. pulmonaria* thalli to genepools and identifying recent migrants, a Bayesian analysis of population structure was run as implemented in STRUCTURE version 2.3.2 (Pritchard et al. 2000). Here, individual multilocus genotypes are probabilistically assigned to a user-defined number of clusters (*K*) assumed to be in gametic equilibrium (i.e., genepools). We ran three replicate simulations for each *K* = {1,…, 15}, and, after a burn-in period of 1 × 10^5^ iterations, 1 × 10^6^ iterations were run in order to sample the posterior distribution. An admixture model was used in which the fraction of ancestry from each genepool was estimated for each sampled individual. The prior of individual admixture was set to be uniform for all genepools. Panmictic genepool allele frequencies were assumed independent of each other (Falush et al. 2003). Individuals (populations) of over 90% (75%) probability of ancestry in a given genepool were regarded as ‘assigned’ to this genepool, whereas all other units (individuals or populations) were classified as ‘admixed’. We inferred the number of genepools by visual inspection of the bar plots at *K* ≥ 2 and by using the methods given in Evanno et al. (2005). The statistic Δ*K* was calculated, which is based on the rate of change in the log likelihood of the data between successive *K* values. The true number of genepools is determined as the modal value of the distribution of Δ*K* over *K* (Evanno et al. 2005). Analyses were run with a data set that included clones (1094 individuals) and with a data set that was corrected for clones within populations (441 individuals).

For a detection of boundaries to gene flow in the study region, we used the Monmonier algorithm (Monmonier 1973) implemented in BARRIER version 2.2 (Manni et al. 2004). As input coordinates, the central coordinates of each population were used (Table 1). In order to detect barriers, pairwise *F*_ST_ values were calculated with 100 bootstrap matrices in MICROSAT version 1.5 (Minch 2001) according to details specified in Werth et al.(2007). Significance levels of pairwise FST values were calculated based on *F*_ST_ *P*-values obtained with Arlequin 3.5.1.2 (Excoffier and Lischer 2010), which were Bonferroni corrected in R (R Development Core Team 2010). We calculated three barriers for each of the 100 bootstrapped matrices.

Average number of alleles (Na), effective mean number of alleles (Ne) and percentage of different genotypes (M) were calculated with Genalex (Peakall and Smouse 2006). Nei's unbiased gene diversity H (Nei 1978) was calculated using the codes written by Werth et al. (2006) in R (R Development Core Team 2010). Measures of allelic richness (A) were corrected for variation in sample size by setting the reference population size equal to the minimal sample size (17) using the rarefaction method as implemented in the software MSA (Dieringer and Schlötterer 2003).

Hierarchical analyses of molecular variance (AMOVA) based on the number of different alleles were calculated with Arlequin 3.5.1.2 (Excoffier and Lischer 2010).

Analyses of variance and one-way ANOVA examining Nei's gene diversity H with categorical, environmental variables were performed with JMP 8.0.2 ([Bibr b50]), as well as tests of categorical models. A general linear model was run to test for the effect of forest state (primeval/managed) on Nei's gene diversity, adjusting for the covariate genepool. Because the primeval forests were not equally distributed over the study region in categorical models, the variable primeval forest was nested within country. The association between genepool and ecological group of tree species was calculated with the G2 likelihood-ratio chi-squared test as implemented in JMP 8.0.2 ([Bibr b50]). Three tree species groups were defined based on distributional and ecological properties. Mesophytic tree species include *A. pseudoplatanus, Pyrus pyraster* and *F. excelsior* and are distributed in humid forests in Central Europe or in ravine forests in (sub) mediterranean Europe. Submediterranean tree species such as *Carpinus orientalis, Castanea sativa, Quercus* sp., and *Quercus frainetto* have their distribution center in Mediterranean mountain regions and warmer areas in Central Europe. *Fagus sylvatica* was the most frequent tree species and was treated as a separate group.

To test for spatial genetic structure in *L. pulmonaria* within the stands and if the structure differed between forest conservation categories, we calculated variograms for genotype diversity for managed and primeval forests based on the method described in Wagner et al. (2005) using R-scripts developed by Werth et al. (2006).

## Results

We analyzed 1094 specimens from 24 populations (Table 1) with eight fungus-specific microsatellite markers. All loci were variable and revealed a total of 190 alleles. A minimum of three different alleles was found in LPu24 and a maximum of 76 alleles were detected in LPu25 (Table A1).

### Inference of population structure

**Figure 2:**
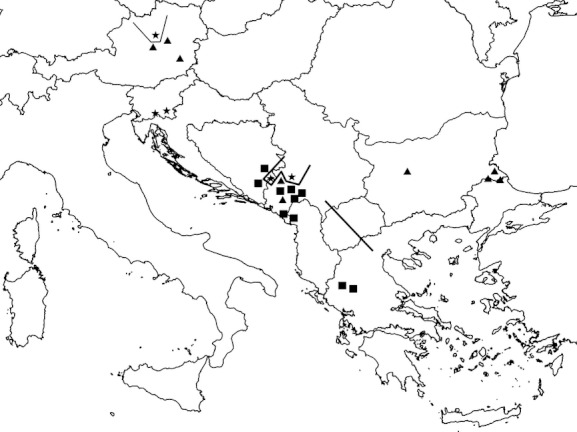
Membership of populations of *Lobaria pulmonaria* to genepool A (triangle), admixed populations (asterisk), and genepool B (quadrat) for *K* = 2 (K2).Genetic discontinuities between 24 populations are indicated by lines. Based on 100 bootstrap replicates, barriers were drawn with line sizes proportional to their bootstrap support (**−** >50<85, **−** >85<100; Manni et al. 2004).

**Figure A1:**
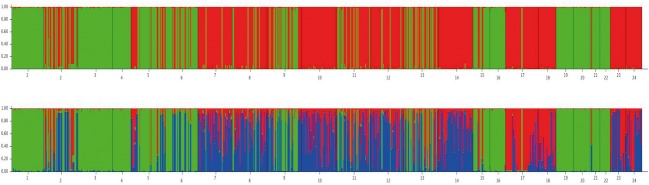
Bar plots of Bayesian analysis of population structure with user-defined number of clusters K = 2 (above) and K = 3 (below). Numbers denote the number of populations: AU5 (1), AU6 (2), AU7 (3), AU8 (4), SL8 (5), SL9 (6), BH1 (7), BH2 (8), BH3 (9), MN1 (10), MN2 (11), MN3 (12), MN4 (13), MN5 (14), MN6 (15), MN7 (16), MN8 (17), MN9 (18), BG1 (19), BG2 (20), BG3 (21), BG4 (22), GR9 (23), GR10 (24).

The Log probability of data L(*K*) strongly increased between *K* = 1 and *K* = 2 and it reached a plateau between *K* = 2 and *K* = 5 before it decreased up to *K* = 15. The highest value of Δ*K* as a function of *K* was reached at *K* = 2 (Fig. A1). The analyses of the data sets with and without clones gave the same results. Visual inspections of the bar plots provided by STRUCTURE confirmed two genepools because at *K* > 2 one of the genepools split in fully admixed populations. Further analyses were therefore carried out assuming the presence of two distinct genepools. The analysis of the inferred ancestry of individuals resulted in 1088 individuals belonging either to genepool A or to genepool B. Only six specimens from six populations (AU5, AU6, MN1, MN2, MN3, MN8) revealed a mixed ancestry (proportion of membership of each predefined population in each of the genepools >0.25<0.75) and only one specimen from the population AU6 contributed equally to the two genepools (0.495 and 0.505, respectively). At the population level 9 and 10 populations belonged to genepools A and B (less than 15% contribution of the alternative genepool), respectively, and five populations revealed admixture to varying degrees (Table 2). Genepool A showed a broad geographic distribution in the area. It was found in the Eastern Alps, in the two canyons along rivers in Montenegro, and in the Central Balkan and the Strandzha Mountains in Bulgaria (Fig. 2). Its altitudinal distribution was concentrated at low altitudes between 300 and 700 m a.s.l. (Table 3).Genepool B was concentrated in Greece, the Coastal Dinarides of Montenegro and Bosnia Herzegovina that is in the primeval beech forest areas of Biogradska gora and Perućica (Fig. 2). Its altitudinal distribution ranged between (480) 1103 and 1613 ma.s.l. which was significantly higher than the mean elevation of genepool A (Table 3). Admixed populations were found in floodplain forests along the rivers in Biogradska gora and Perućica, the two Slovenian populations and in the primeval forest “Rotwald” in the northern Alps (AU6), which has a 40% membership to genepool A. The altitudinal distribution of admixed populations ranged from 829 to 1104 ma.s.l., their average altitude being significantly lower than genepool A, but not different from genepool B (Table 3).

**Table 2:** Studied populations of *Lobaria pulmonaria* with number of samples (N), average number of alleles (Na), effective mean number of alleles (Ne), average allelic richness (A), percentage of different genotypes (M), Nei's unbiased gene diversity (H), average number of private alleles (P), the proportion of membership of populations in genepool A for *K* = 2 (K2) and the membership to genepool A or B or of admixed (A/B) nature

Population	N	Na	Ne	A	M	He	H	LC	K2	Genepool
AU5	55	5.13	3.26	4.40	0.55	0.45	0.46	2.88	0.99	A
AU6	60	7.50	3.65	5.79	0.58	0.56	0.57	3.75	0.41	A/B
AU7	60	5.63	3.49	4.63	0.55	0.45	0.46	3.13	0.99	A
AU8	32	3.75	2.49	3.51	0.50	0.38	0.40	1.75	0.99	A
SL8	60	7.75	3.76	6.31	0.50	0.58	0.59	4.38	0.58	A/B
SL9	56	6.38	3.65	5.47	0.72	0.57	0.58	2.88	0.73	A/B
BH1	60	6.50	3.25	5.17	0.59	0.49	0.51	3.75	0.05	B
BH2	60	7.00	3.41	5.58	0.72	0.50	0.51	4.13	0.14	B
BH3	60	8.13	3.89	6.37	0.77	0.61	0.62	5.00	0.46	A/B
MN1	60	7.75	4.36	5.75	0.83	0.51	0.52	4.38	0.01	B
MN2	60	9.13	5.88	6.93	0.80	0.58	0.59	5.13	0.23	B
MN3	58	6.75	4.06	5.57	0.82	0.53	0.54	3.75	0.08	B
MN4	60	9.38	5.15	7.01	0.50	0.61	0.62	5.38	0.39	A/B
MN5	60	6.50	4.04	5.21	0.85	0.49	0.50	4.00	0.01	B
MN6	29	5.13	3.24	4.89	0.90	0.51	0.53	2.25	0.76	A
MN7	27	4.63	3.12	4.43	0.78	0.39	0.41	2.25	0.99	A
MN8	57	6.00	2.66	4.74	0.83	0.46	0.47	3.63	0.04	B
MN9	31	6.00	3.63	5.49	0.72	0.51	0.53	3.00	0.01	B
BG1	30	4.50	2.77	4.25	0.55	0.47	0.48	2.13	0.99	A
BG2	29	4.13	2.57	3.94	0.82	0.40	0.42	2.25	0.99	A
BG3	17	4.00	2.97	4.00	0.35	0.53	0.56	2.13	0.88	A
BG4	18	4.25	2.78	4.25	0.71	0.43	0.45	2.50	0.99	A
GR9	27	4.00	2.58	3.87	0.52	0.47	0.51	2.00	0.01	B
GR10	28	5.00	3.54	4.77	0.46	0.49	0.52	2.75	0.01	B

**Table 3:** Analysis of variance and average values of groups in one-wayANOVA examining the altitudinal distribution [m] of gene pools A, B, and admixed in *Lobaria pulmonaria* from southeastern Europe. SE uses a pooled estimate of error variance. Levels of Tukey-Kramer HSD *t*-test with different letters are significantly different from one another

Source	df	Sum of squares	Mean square	*F*	*P*	
Genepool	2	2,458,979.100	1,229,489.550	15.569	0.0001	
Error	21	1,658,357.520	78,969.406			
Total	23	4,117,336.630				

Level	Number	Mean	SE	Lower 95%	Upper 95%	*t*-test
Genepool A	9	496.444	93.672	301.644	691.245	A
Admixed	5	950.800	125.674	689.447	1212.153	B
Genepool B	10	1213.500	88.865	1028.696	1398.304	B

Using the Monmonier algorithm (Manni et al. 2004), we found regional genetic discontinuities with high bootstrap support (Fig. 2). In the Central Dinarides the two populations collected in the canyons Tara (MN7) and Mrtvica (MN6) revealed clear discontinuities to the primeval forest areas Perućica (BH1-3) in the northeast and the Biogradska gora national park in the southeast, and the coastal Dinarides with the mountain system Rumija (MN8-9) in the southwest. The Greek populations were discontinuous with the Bulgarian populations. Also within the Austrian Alps the Rotwald virgin forest (AU6) was distinct from the surrounding populations, including those in Slovenia (Fig. 2).

### Genetic differentiation

Most of the 300 pairwise comparisons of populations showed significant pairwise FST values. If clones were included in the datasets (recurring genotypes, 1094 samples) 252 pairs (84%) and in the clone-corrected dataset (441 samples) 138 pairs (46%) revealed significant levels of genetic differentiation (Table A2).

The AMOVA analyses revealed that only 8.91% of the genetic variation of the clone-corrected dataset was explained by the five biogeographic regions Alps, Dinarides, Strandzha, Balkan- and Pindus Mountains and the fixation index *F*_ST_ of the complete data set that included clones was 0.143 compared to 0.198 of the clone-corrected dataset (data not shown). *Lobaria pulmonaria* populations growing on *F. sylvatica* were not differentiated from populations growing on other tree species (1.25%, *P* > 0.104) and populations from managed forests were not differentiated from those of primeval forests (1.77% differentiation, *P* > 0.074).

### Genetic diversity

Because some populations were small and only scarce material of the rare and locally threatened lichen species was found, it was not possible to collect the planned 60 specimens in each population. To avoid damage on small populations, less than the planned number of specimens was collected in 14 of the 24 populations (Table 2). The number of collected specimens N was positively correlated with Na, Ne but N had no significant effect on Nei's unbiased gene diversity H and the percentage of different multilocus genotypes (M).

**Figure 3:**
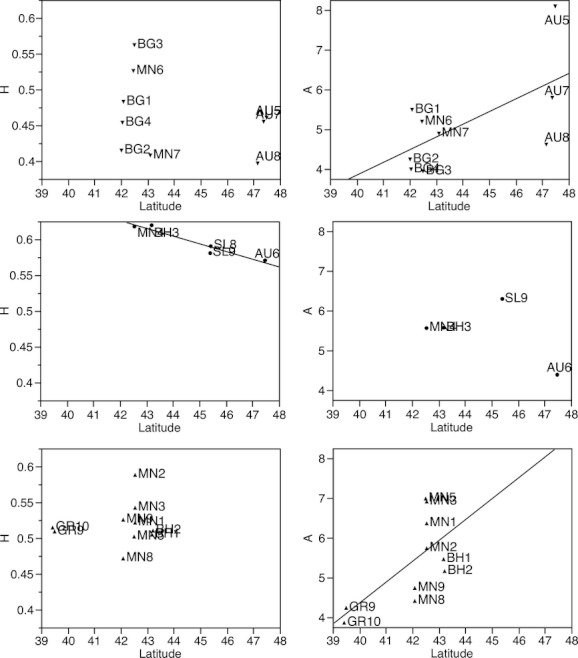
Linear fit of Nei's unbiased gene diversity H (left) and allelic richness A (right) with latitude. (*P*-values for slopes = 0) for genepool A (inverted triangle, H: *P* < 0.71, A: *P* < 0.07), admixed populations (circle, H: *P* < 0.008, A: *P* < 0.44), and genepool B (triangle, H: *P* < 0.37, A: *P* < 0.042).

Nei's unbiased gene diversity H was significantly higher in populations that belonged to genepool B than to genepool A and the highest values were reached in admixed populations (Table 4). For the latter we found a significant decrease in Nei's unbiased gene diversity H with increasing latitude, indicating spread from a southern refuge. No latitudinal gradients could be found for the two genepools A and B, respectively, but the admixed populations showed a decreasing gene diversity with increasing latitude. The average allelic richness of genepool A (*P* < 0.07) and B (*P* < 0.04) was positively correlated with latitude (Fig. 3).

**Table 4:** Analysis of variance and average values of groups in one-way ANOVA examining Nei's unbiased gene diversity H in *Lobaria pulmonaria* from southeastern Europe. Standard error uses a pooled estimate of error variance. Levels of Tukey-Kramer HSD *t*-test with different letters are significantly different from one another

Source	df	Sum of squares	Mean square	*F*	*P*	
Genepool	2	0.058	0.029	17.787	0.00003	
Error	21	0.034	0.002			
Total	23	0.093				

Level	Number	Mean	SE	Lower 95%	Upper 95%	*t*-test
Genepool A	9	0.463	0.013	0.435	0.491	A
Admixed	5	0.597	0.018	0.559	0.634	B
Genepool B	10	0.520	0.013	0.493	0.546	C

Nei's unbiased gene diversity H was also significantly higher in primeval forest reserves than in managed forests when considering all populations (data not shown). Because primeval forest reserves were not equally distributed over the entire study area we also tested this difference for the 13 populations analyzed in Montenegro and Austria where primeval and managed forests were found closely together, and were able to confirm statistically higher H values for the primeval forest reserves (Table 5).

**Table 5:** Analysis of variance and average values of groups in one-way ANOVA examining Nei's unbiased gene diversity H in 13 populations from countries with populations from both primeval and managed forests (Austria and Montenegro) for *Lobaria pulmonaria* in southeastern Europe. SE uses a pooled estimate of error variance. Levels of Tukey-Kramer HSD *t*-test with different letters are significantly different from one another

Source	df	Sum of squares	Mean square	*F*	*P*	
Forest protection status	1	0.0309	0.0309	15.0810	0.0025	
Error	11	0.0225	0.0020			
Total	12	0.0534				

Level	Number	Mean	SE	Lower 95%	Upper 95%	*t*-test
Primeval forest reserve	5	0.5689	0.0202	0.5243	0.6134	A
Managed forest	8	0.4687	0.0160	0.4334	0.5039	B

**Figure 4:**
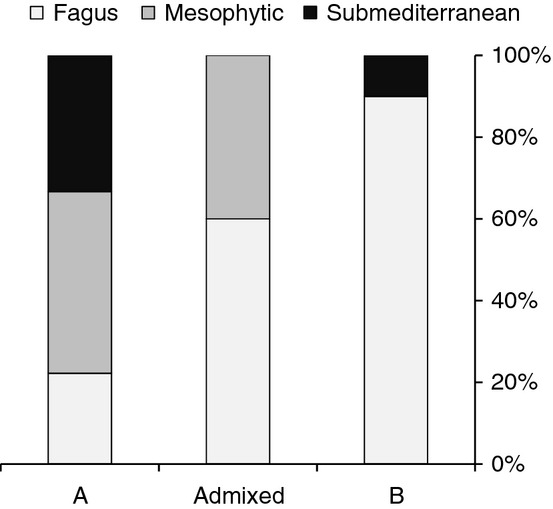
Association of *Lobaria pulmonaria* populations belonging to genepools A, admixed and B with their carrier tree species groups. The association is statistically significant (likelihood-ratio χ^2^ = 13.73, *P* < 0.008; Pearson χ^2^ = 10.64, *P* < 0.031).

The most frequent carrier tree species of *L. pulmonaria* in South Eastern Europe was *F. sylvatica*, but *F. excelsior*, *A. pseudoplatanus*, *Quercus* sp. played a role in floodplain forests and *A. cephalonica* was a frequent habitat in Greece. Although we did not find significant association between individual tree species and genepools, our dataset revealed significant association of genepools with three tree species groups with different ecological preferences (Fig. 4, Table 1). Both genepools were found with submediterranean tree species and with *Fagus*, although at different proportions. About 30% of the populations of genepool A were associated with submediterranean tree species, 40% with mesophytic tree species and only 20% were associated with beech, whereas 90% of the populations belonging to genepool B were associated with *Fagus* and one population was growing on *Castanea sativa*, a submediterranean species. The admixed populations were found mostly on beech or on mesophytic tree species (Fig. 4).

### Spatial analysis of genotype diversity

**Figure 5:**
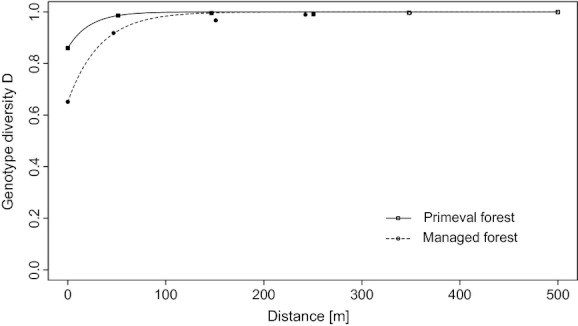
Semivariogram of genotype diversity (D) in *Lobaria pulmonaria*. Each symbol indicates the probability of sampling two different multilocus genotypes as a function of their distance in space. Squares, black line: primeval forests; triangles, dotted line: managed forests. Filled symbols indicate significant spatial autocorrelation.

Pairwise genotype diversities between thalli were significantly reduced below distances of 250 m. At small distances up to 170 m genotype diversity was lower in managed forests compared with primeval forests (Fig. 5). Distance class 0, i.e. specimens collected from the same tree, pairwise genotype diversities revealed a substantially lower genotype diversity in managed forests than in primeval forests, even though our sampling design was chosen to minimize sampling the same genet repeatedly on a tree.

## Discussion

We investigated the population genetic structure of the tree-colonizing lichen species *Lobaria pulmonaria* in the Eastern Alps and South Eastern Europe by measuring variation at eight microsatellite loci. We collected population data from primeval forest reserves and compared their genetic diversity with populations from extensively managed forests. We also made inference about patterns of postglacial recolonization in South Eastern Europe and tested the association of genepools with forest-tree species groups.

### Genepools

In South Eastern Europe two distinct genepools and admixed populations were identified in our study. Genepool B shows a strictly Mediterranean and Sub Mediterranean distribution pattern and contrasts with Genepool A, which showed a wide geographic distribution from the northern slope of the Alps, to the river valleys in Montenegro, the Central Balkan range and Strandzha Mountains in Bulgaria. Populations with >15% admixture were found in the primeval forest reserve Rotwald in the Eastern Alps, in the Slovenian Dinarides as well as between populations of either genepool in the Central Dinarides. Despite the frequent admixture in populations only one individual was found with equally strong relations to the two genepools. This individual was found in the population AU6 where the two genepools were equally frequent and spatially mixed in a relatively dense population. This individual can thus be considered as offspring of a mating event between parents belonging to the two respective genepools. Mating events between genepools seem to be rare in admixed populations despite the fact that about 70% of the populations studied included fertile individuals and despite the expected heterothallic mating system of *L. pulmonaria* (Zoller et al. 1999). A recent analysis revealed that a conservative estimate of 7.7% of the pairwise comparisons of the mycobionts were the result of recombinations and/or relichenization (Dal Grande et al. 2012) in *L. pulmonaria*. Our results revealed an even lower percentage of mating between genepools. Thus, further studies will have to shed light on possible reproductive barriers between genepools of this lichen species. Low percentage of different genotypes M found in many populations point toward the key importance of clonality as reproductive mode (Dal Grande et al. 2012; Werth and Scheidegger 2012).

Pairwise *F*_ST_ of populations corroborate the two genepools detected by the Bayesian analyses. They correspond to two of the four European genepools that were identified in *L. pulmonaria* in a recent analysis (Widmer 2009). Similarly, earlier studies by Walser and co-workers revealed genetic differentiation between inland and coastal populations in British Columbia, indicating multiple postglacial recolonization pathways within this region (Walser et al. 2005). The same authors showed genetic isolation effects between North American and European populations (Walser et al. 2005).

### Phylogeography

High allelic richness is characteristic of refugial areas and of contact zones, the geographic areas where lineages expanding from different refugia meet (Widmer and Lexer 2001). Genetic diversity was higher in populations belonging to genepool B than those that belonged to genepool A. The highest levels of gene diversity were found in populations of an admixed nature, confirming that admixture effects can lead to a secondary increase in diversity (Comps et al. 2001). We found a significant effect of latitude on allelic richness in genepool B and marginal significance in genepool A, but not in the admixed populations. In genepool B, the highest values of allelic richness were found in the northeastern populations of Montenegro, whereas the southern populations in Greece showed lower allelic richness.

Genepool B dominated in Southeast European mountain forests above 1000 ma.s.l. concentrated in the extended beech-forest areas including the primeval forests areas of Perućica and Biogradska gora. However, populations consisting of pure genepool B were not found in Slovenia, and despite its abundance in southern Bosnia and Herzegovina we found no evidence that pure populations of this genepool contributed to the recolonization of the Eastern Alps. Three of four studied populations from the Eastern Alps belonged to genepool A and shared stronger relations to populations from the Central Balkan and the Bulgarian Black Sea coast compared with geographically closer populations in Slovenia. The allelic richness of genepool A was positively correlated with latitude, reaching its highest values in the Alps. This is evidence of a northern refuge of *L. pulmonaria* in the Alps or in the Carpathians (Nadyeina et al. 2012) rather than in more southeastern regions such as Bulgaria. Furthermore, two populations belonging to this genepool were found in forests along rivers in the Central Dinarides. Thus, migration routes of genepool A likely followed river valleys, which are supported by the lower altitudinal distribution of genepool A compared with genepool B. Also populations consisting of a combination of genepools A and B were found in several regions. The southernmost locality of an admixed population was found in the Central Dinarides, that is, where both genepools A and B were found within short distance. Admixed populations in the Central Dinarides were confined to mountain lakes, and are thus geographically and ecologically related to the expected migration routes along rivers as described for genepool A.

### Hypothetical scenario of postglacial colonization

Although in regions with high air humidity the epiphytic *L. pulmonaria* also rarely occurs on rocks (Rose and Purvis 2009), saxicolous populations in Central and Southern Europe can be interpreted as sink populations depending on neighboring epiphytic, forest-bound source populations. This lichen's postglacial colonization therefore depends on forest-tree refuges.

We found significant association between three ecologically defined groups of tree species (*Fagus*; temperate deciduous and submediterranean deciduous trees) and genepools, which may indicate “hitchhiking” of *L. pulmonaria* on forest communities during postglacial migrations. However, we did not find an association with individual tree species, which indicates that frequent switches of carrier tree species within ecological groups have occurred.

Genepool B of *L. pulmonaria* is associated with *F. sylvatica* and we can hypothesize genepool B survived the last glaciation associated within the refuge of *F. sylvatica* on the Coastal and Central Dinarides (Magri et al. 2006). With the southward migration of isozyme group A of *Fagus* (Magri et al. 2006) genepool B of *L. pulmonaria* likely spread southwards from the northern part of the Dinarides. This is supported by decreasing allelic richness from the northern part of the Dinarides toward the South. However, the fact that Nei's gene diversity was not decreasing with latitude indicates that postglacial migration distances were small. It is therefore likely that in the southern part of the study area, genepool B was associated with additional tree species including *A. cephalonica*.

Genepool A has a wider geographic distribution compared with genepool B. All over this area, Nei's gene diversity was not significantly changing along the long latitudinal and longitudinal gradients. We interpret this result as evidence for the existence of multiple refugia including the Black Sea Coast near the Strandzha Mountains, the Balkan Mountains, ravine forests in the Central Dinarides and possibly the South Eastern Alps. Because temperature anomalies in Southeastern Europe were small compared with Southwestern or Central and Northern Europe (Davis et al. 2003), early Holocene distribution of *L. pulmonaria* was possibly limited by aridity and the lack of suitable carrier trees, rather than by temperature. Elevated levels of air humidity, which is more important than precipitation for this poikilohydric lichen (Scheidegger et al. 1995; Pannewitz et al. 2003) were available within the abundant karstic depressions in these regions and along rivers. Also, these regions are known refugial areas for various important carrier trees of *L. pulmonaria* including *A. alba* (Linares 2011), *F. excelsior* (Heuertz et al. 2004), and deciduous oaks (Brewer et al. 2002). Additionally, to a refuge of *F. sylvatica* on the Coastal and Central Dinarides (Magri et al. 2006) there is also growing evidence for refuges of European beech in the southeastern Alps (Brus 2010) where genepool A was abundant and reached equally high levels of Nei's gene diversity as, and higher levels of allelic richness than, the more southern and eastern populations.

Taking into account the current distribution of genepool A in ravine forests along river valleys such as Tara and Mrtvica in Montenegro we hypothesize a Central Dinarides refuge of *L. pulmonaria* associated with mesophytic tree species such as *F. excelsior* (Heuertz et al. 2004). In the Central Dinarides, we found the two genepools in close proximity but ecologically separated into a beech-forest-associated genepool B and a ravine-forest-associated genepool A. Although the two genepools are characterized with neutral markers, it is likely that populations belonging to the respective genepools will reveal ecological differentiation, in addition to the abovementioned altitudinal differentiation of genepools A and B. Our data do not allow to disentangle the complex interactions between altitude, climate (and paleoclimate), and tree species groups with the current genetic pattern. Future studies should therefore look at adaptive genetic variation of both photobiont and mycobiont to see if these neutral gene pool variants might be associated with genes or genetic variants of adaptive relevance.

Unlike the two genepools A and B where allelic richness and latitude are positively correlated, admixed populations showed a significant decrease in Nei's gene diversity with increasing latitude. The highest values of Nei's gene diversity were found in western Montenegro and eastern Bosnia and Herzegovina where suture zones between genepools A and B were found in our study. Decreasing gene diversity with latitude indicates postglacial spread over larger distances from a refugial area of admixed populations. Because no pure populations belonging to genepool B could be found in the Alps, it is likely that admixture of the two genepools occurred prior to postglacial spread. The data available so far even point to the possibility that the admixed genepool survived the last glacial maximum in Slovenia and the Eastern Alps. Because our results indicate an independent glacial history of the admixed populations, we can conclude that the distinct genepools A and B reflect an evolutionary history longer than the last glacial–interglacial cycle. The Quaternary history of this epiphytic lichen species thus suggests that the modern genetic diversity is likely the result of multiple interglacial–glacial cycles, possibly since the middle Pleistocene (Magri et al. 2006; Magri 2010). The Quaternary history of the lichen thus parallels its carrier tree species, especially the European beech (Magri et al. 2006; Brus 2010). Similar to parasitic or mutualistic symbioses an epiphytic organism may thus show phylogeographic congruence with its carrier tree.

### Conservation value of primeval forests

*Lobaria pulmonaria* populations can be maintained in managed forests only if management intensity is low and a large portion of retention trees carrying this lichen are maintained during forest management (Scheidegger et al. 1998, 2000; Wagner et al. 2006; Werth et al. 2006, 2007; Scheidegger and Werth 2009).Our studies showed that primeval forest areas with a strict protection status harbor populations with a higher genetic diversity than managed forests. Thus, even low levels of forest management significantly reduce genetic diversity of *L. pulmonaria*, long before the abundance of this species might be negatively affected. This confirms earlier studies on effects of disturbance gradients on genetic diversity of this lichen species. Werth et al. (2007) found reduced genetic diversity of *L. pulmonaria* in a regenerating stand after a forest fire, compared with stands, which were managed by single-stem harvest since early medieval times. In Estonia *L. pulmonaria* had a higher genetic diversity and more juvenile thalli were found in old-growth forests compared with managed forests and wooded meadows (Jüriado et al. 2011). Because recent fragmentation of a large, previously unmanaged forest landscape did not directly affect genetic diversity of its *L. pulmonaria* population (Otalora et al. 2011), loss of genetic diversity in *L. pulmonaria* is likely the result of genetic drift after significant reduction of regional population size and fragmentation into small, spatially isolated populations. Because genetic drift accumulates over many generations (Frankham et al. 2002), loss of genetic diversity is a creeping consequence of continuing forest management.

A comparison of the spatial genetic diversity between managed and protected primeval populations revealed that genotype diversity was substantially lower up to 170 m in managed forests compared with primeval forests. With a reduced genotype diversity at low distances in managed forests we conclude that while clonal spread is dominant at low distance classes (Dal Grande et al. 2012; Werth and Scheidegger 2012) fewer genotypes are spatially mixed within their dispersal range in managed compared with primeval forests. Because *L. pulmonaria* is a heterothallic species (Zoller et al. 1999) intra-haploid mating is prevented (Billiard et al. 2011) and sexual reproduction is only possible when genetically different, compatible multilocus genotypes grow in close vicinity. Although population density may be high also in managed forests, if appropriate conservation measures such as retention trees for biodiversity conservation are implemented, harvesting mature trees inevitably increases the average distance between carrier trees of *L. pulmonaria*. In general an increased average distance between carrier trees will reduce gene flow and thus lower the genetic diversity of *Lobaria* at the tree level. Only primeval forests and forest reserves can maintain the required small-scale connectivity of habitats over centuries and thus facilitate genetically diverse *L. pulmonaria* populations.

Large-scale primeval forest reserves can thus be considered reference systems for characteristic levels of genetic diversity of populations of *L. pulmonaria* and possibly other old-growth forest dependent species. Therefore, we are concerned that none of the primeval forests harbored genepool A of *L. pulmonaria*. All populations belonging to genepool A were found in currently or previously managed forest stands and deserve high conservation priority in future.

## Appendix

**Appendix: Table A1:** Number of alleles per each microsatellite marker and population in *Lobaria pulmonaria*

Population	LPu03	LPu09	LPu15	LPu23	LPu24	LPu25	LPu28	MS4	All
A5	3	5	6	1	1	17	4	4	41
A6	4	7	11	3	1	17	13	4	60
A7	3	4	8	1	1	21	4	3	45
A8	2	4	4	1	1	13	2	3	30
SL8	4	8	11	3	1	17	12	6	62
SL9	4	11	8	2	1	15	6	4	51
BH1	2	3	8	4	1	15	15	4	52
BH2	3	5	10	3	1	17	12	5	56
BH3	4	6	11	3	1	21	13	6	65
MN1	2	6	7	2	1	27	14	3	62
MN2	3	6	10	3	1	28	19	3	73
MN3	3	4	6	3	1	22	12	3	54
MN4	3	11	11	3	1	27	14	5	75
MN5	2	3	7	2	1	23	11	3	52
MN6	2	10	6	3	1	10	6	3	41
MN7	2	7	6	1	1	14	3	3	37
MN8	1	3	8	4	2	15	11	4	48
MN9	1	5	10	2	2	14	10	4	48
BG1	2	7	5	2	1	10	5	4	36
BG2	3	6	5	1	1	11	3	3	33
BG3	3	5	6	2	1	8	3	4	32
BG4	2	7	5	1	2	11	4	2	34
GR9	1	3	7	2	1	8	7	3	32
GR10	1	3	7	2	1	13	10	3	40
All	4	34	24	5	3	76	31	13	

**Appendix: Table A2:** Pairwise *F*_ST_ values of populations with recurrent multilocus genotypes (above diagonal) and without recurrent genotypes (below diagonal). Significant values are printed in bold

	AU5	AU6	AU7	AU8	SL8	SL9	BH1	BH2	BH3	MN1	MN2	MN3	MN4	MN5	MN6	MN7	MN8	MN9	BG1	BG2	BG3	BG4	GR9	GR10
AU5	0.000	**0.170**	**0.035**	0.060	**0.100**	**0.082**	**0.150**	**0.154**	**0.086**	**0.042**	**0.094**	**0.059**	**0.060**	**0.041**	**0.322**	**0.295**	**0.322**	**0.216**	**0.305**	**0.339**	**0.360**	**0.339**	**0.358**	**0.347**
AU6	0.025	0.000	**0.159**	**0.185**	**0.051**	**0.076**	**0.063**	**0.063**	**0.118**	**0.200**	**0.175**	**0.182**	**0.128**	**0.170**	**0.107**	**0.103**	**0.135**	**0.073**	**0.102**	**0.134**	**0.197**	**0.113**	**0.148**	**0.147**
AU7	−0.025	**0.056**	0.000	**0.038**	**0.088**	**0.062**	**0.138**	**0.144**	**0.103**	**0.072**	**0.084**	**0.082**	**0.056**	**0.087**	**0.305**	**0.278**	**0.309**	**0.203**	**0.287**	**0.326**	**0.337**	**0.321**	**0.337**	**0.326**
AU8	0.009	0.056	0.046	0.000	**0.100**	**0.069**	**0.132**	**0.153**	**0.102**	**0.069**	**0.127**	**0.090**	0.089	**0.091**	**0.314**	**0.283**	**0.322**	**0.210**	**0.298**	**0.339**	**0.348**	**0.337**	**0.364**	**0.345**
SL8	0.052	0.005	**0.088**	0.033	0.000	**0.036**	**0.032**	**0.049**	**0.051**	**0.100**	**0.115**	**0.093**	0.056	**0.087**	**0.145**	**0.118**	**0.159**	**0.079**	**0.130**	**0.165**	**0.205**	**0.156**	**0.171**	**0.166**
SL9	−0.002	0.010	0.032	0.005	0.005	0.000	**0.063**	**0.065**	**0.061**	**0.104**	**0.091**	**0.105**	**0.070**	**0.085**	**0.175**	**0.160**	**0.193**	**0.103**	**0.164**	**0.200**	**0.241**	**0.192**	**0.212**	**0.207**
BH1	**0.236**	**0.112**	**0.279**	**0.246**	**0.089**	**0.157**	0.000	0.033	**0.078**	**0.169**	**0.160**	**0.150**	0.068	**0.138**	**0.073**	**0.051**	**0.099**	**0.045**	**0.071**	**0.103**	**0.155**	**0.120**	**0.106**	**0.095**
BH2	**0.171**	**0.069**	**0.211**	**0.157**	0.030	**0.097**	−0.006	0.000	**0.063**	**0.167**	**0.145**	**0.158**	**0.102**	**0.138**	**0.081**	**0.077**	**0.063**	0.012	**0.058**	**0.093**	**0.150**	**0.111**	**0.095**	**0.083**
BH3	0.058	0.014	**0.095**	0.036	−0.010	0.009	**0.075**	0.021	0.000	**0.098**	**0.097**	**0.092**	**0.073**	0.047	**0.210**	**0.186**	**0.210**	**0.116**	**0.193**	**0.232**	**0.257**	**0.227**	**0.236**	**0.227**
MN1	**0.290**	**0.165**	**0.334**	**0.309**	**0.141**	**0.215**	0.016	0.026	**0.132**	0.000	**0.112**	0.028	**0.097**	0.047	**0.359**	**0.332**	**0.349**	**0.237**	**0.334**	**0.372**	**0.399**	**0.377**	**0.411**	**0.392**
MN2	**0.166**	**0.076**	**0.209**	**0.164**	**0.042**	**0.100**	0.006	−0.006	0.037	0.019	0.000	**0.109**	**0.095**	**0.086**	**0.307**	**0.285**	**0.300**	**0.210**	**0.286**	**0.326**	**0.354**	**0.323**	**0.334**	**0.328**
MN3	**0.183**	0.073	**0.225**	**0.182**	0.040	0.104	−0.007	−0.015	0.036	0.023	−0.010	0.000	0.064	0.025	**0.342**	**0.321**	**0.347**	**0.237**	**0.319**	**0.364**	**0.397**	**0.372**	**0.395**	**0.381**
MN4	**0.093**	0.029	**0.127**	**0.086**	0.009	**0.043**	**0.044**	0.006	0.004	**0.062**	0.002	0.012	0.000	**0.066**	**0.243**	**0.214**	**0.246**	**0.153**	**0.221**	**0.262**	**0.294**	**0.258**	**0.272**	**0.263**
MN5	**0.280**	**0.146**	**0.323**	**0.295**	**0.114**	**0.193**	0.014	0.019	**0.111**	0.021	0.017	−0.004	0.072	0.000	**0.325**	**0.298**	**0.317**	**0.207**	**0.296**	**0.335**	**0.365**	**0.329**	**0.362**	**0.344**
MN6	0.022	0.032	0.061	0.027	0.027	0.012	**0.185**	**0.119**	0.041	**0.236**	**0.115**	0.132	0.044	**0.225**	0.000	0.010	**0.052**	**0.052**	**0.023**	**0.060**	**0.136**	**0.076**	**0.051**	**0.058**
MN7	0.054	**0.100**	**0.099**	0.015	**0.082**	0.042	**0.289**	**0.212**	**0.082**	**0.346**	**0.210**	**0.237**	**0.125**	**0.345**	**0.066**	0.000	**0.048**	**0.041**	**0.025**	**0.039**	**0.125**	**0.067**	**0.031**	0.029
MN8	**0.263**	**0.136**	**0.303**	**0.262**	**0.099**	**0.179**	0.003	0.001	**0.091**	0.008	0.010	0.013	**0.049**	0.016	**0.195**	**0.322**	0.000	0.018	0.013	0.013	**0.144**	**0.079**	0.035	0.006
MN9	**0.271**	**0.142**	**0.306**	**0.278**	**0.105**	**0.184**	0.029	0.034	**0.107**	**0.071**	0.034	0.037	**0.075**	0.026	**0.208**	**0.330**	−0.001	0.000	0.020	**0.035**	**0.133**	**0.065**	**0.053**	**0.036**
BG1	−0.006	0.034	0.029	0.027	0.049	0.004	**0.241**	**0.169**	0.062	**0.295**	**0.172**	**0.189**	**0.086**	**0.292**	0.006	0.004	**0.266**	0.270	0.000	0.008	**0.157**	**0.062**	0.035	**0.030**
BG2	−0.009	0.055	0.024	−0.001	0.061	0.012	**0.277**	**0.199**	0.066	**0.337**	**0.204**	**0.211**	**0.113**	**0.326**	0.026	−0.003	**0.307**	0.313	−0.030	0.000	**0.173**	**0.065**	0.036	0.020
BG3	−0.005	−0.005	0.044	−0.013	−0.006	−0.013	**0.153**	0.075	−0.010	**0.216**	0.098	0.107	0.028	**0.206**	0.004	0.036	**0.176**	0.192	−0.017	0.004	0.000	**0.122**	**0.147**	**0.118**
BG4	−0.010	0.069	0.050	0.008	0.069	0.033	**0.285**	**0.207**	0.078	**0.339**	**0.203**	**0.220**	**0.115**	**0.327**	0.005	0.027	**0.309**	0.308	−0.018	−0.026	0.010	0.000	**0.075**	**0.073**
GR9	**0.230**	**0.106**	**0.262**	**0.240**	0.088	0.160	0.038	0.023	0.089	0.056	0.030	0.027	0.038	0.048	**0.136**	**0.313**	0.013	**0.026**	**0.233**	**0.277**	**0.154**	**0.265**	0.000	0.011
GR10	**0.263**	**0.136**	**0.297**	**0.271**	**0.099**	**0.182**	0.013	−0.001	**0.093**	0.012	0.016	0.019	0.043	0.030	**0.187**	**0.335**	−0.022	**0.005**	**0.265**	**0.309**	**0.180**	**0.305**	−0.030	0.000
